# Assembly of a Marine Viral Metagenome after Physical Fractionation

**DOI:** 10.1371/journal.pone.0060604

**Published:** 2013-04-08

**Authors:** Jennifer R. Brum, Alexander I. Culley, Grieg F. Steward

**Affiliations:** Department of Oceanography, University of Hawai‘i at Manoa, Honolulu, Hawai‘i, United States of America; Universidad Miguel Hernandez, Spain

## Abstract

Metagenomic analyses of marine viruses generate an overview of viral genes present in a sample, but the percentage of the resulting sequence fragments that can be reassembled is low and the phenotype of the virus from which a given sequence derives is usually unknown. In this study, we employed physical fractionation to characterize the morphological and genomic traits of a subset of uncultivated viruses from a natural marine assemblage. Viruses from Kāne‘ohe Bay, Hawai‘i were fractionated by equilibrium buoyant density centrifugation in a cesium chloride (CsCl) gradient, and one fraction from the CsCl gradient was then further fractionated by strong anion-exchange chromatography. One of the fractions resulting from this two-dimensional separation appeared to be dominated by only a few virus types based on genome sizes and morphology. Sequences generated from a shotgun clone library of the viruses in this fraction were assembled into significantly more numerous contigs than have been generated with previous metagenomic investigations of whole DNA viral assemblages with comparable sequencing effort. Analysis of the longer contigs (up to 6.5 kb) assembled from our metagenome allowed us to assess gene arrangement in this subset of marine viruses. Our results demonstrate the potential for physical fractionation to facilitate sequence assembly from viral metagenomes and permit linking of morphological and genomic data for uncultivated viruses.

## Introduction

Viruses are the most abundant biological entities in aquatic environments and have significant roles that include causing mortality, mediating genetic exchange, and altering the genetic potential of their hosts [1]. Investigations of the morphology (reviewed by [2]) and genome size distributions [3] of aquatic viruses have shown that they are a diverse component of aquatic ecosystems. However, investigating the genomic content of this diverse array of viruses has proven to be challenging.

Isolation of viruses from cultivated hosts allows for the sequencing of complete viral genomes which can be used to connect genomic with phenotypic information (e.g., [4], [5]) and to determine the gene organization and genetic capabilities of a given virus (e.g., [4], [6]). However, the ability to investigate viruses in this way is limited by the requirement of host cultivation. It has been estimated that >99% of environmental microorganisms are uncultivated [7] and that the groups of microorganisms that are in culture may not be representative of the environments from which they originate [8].

This cultivation bottleneck has led to the investigation of viral assemblages using metagenomics, in which random pieces of nucleic acid from viral samples are sequenced, resulting in a survey of viral genes within a sample (reviewed by [9]). Metagenomic analyses have supported the assessment that aquatic viruses are extraordinarily diverse, but the majority of sequences obtained from these investigations are not similar to known genes, indicating that much of the genomic information in aquatic viruses has yet to be characterized [10].

The high diversity of aquatic viral communities means that very few sequences from metagenomic analyses can be reassembled into larger stretches of sequence [11]–[13]. Without reassembly of the fragmented genomes, the genetic structure of individual viruses cannot be assessed and genes cannot be investigated within the context of whole genomes. The current methods used to construct these metagenomic libraries also eliminate any phenotypic information about viruses in the samples.

So far, with the exception of a small single-stranded DNA virus [14], reassembly of uncultivated prokaryotic and viral genomes from shotgun libraries of aquatic assemblages has only been achieved with samples that contain low diversity of bacteria or viruses [15]–[17]. This had led to the suggestion that, in addition to advances in sequencing technology and computational methods [18]–[20], there should also be a focus on improving upstream methods that are used to prepare samples for metagenomic analyses, specifically methods that reduce the diversity of the samples through physical fractionation [21]. In fact, computational models have shown that separating viruses from a sample into two or more fractions can increase the assembly of sequenced DNA fragments from the constituent viral assemblage [22].

Multi-dimensional physical fractionation of natural aquatic viral assemblages can be achieved by exploiting differences in the sizes, surface charges, and buoyant densities among different populations of viruses [23]. Here, we use two physical fractionation steps in series to enrich a limited number of viral consortia from a complex marine assemblage in order to test whether such a procedure would result in a high proportion of assembled sequences.

## Materials and Methods

### Ethics Statement

No specific permits were required for the described field studies. Samples were collected from public waters and no specific permissions were required. Samples consisted of microscopic plankton, which are not endangered or protected.

### Sample Collection

A viral concentrate was collected on October 17, 2006 from a depth of 3 m approximately 25 m off the southeast shore of Coconut Island (Moku O Lo‘e) located in Kāne‘ohe Bay, Oahu, HI. Approximately 1800 l of water was filtered through 0.2 µm pore-size cartridge filters with polyethersulfone membranes (Polycap, Whatman). Viruses in the filtrate were concentrated with a tangential flow filtration cassette with 100 kDa nominal molecular weight cut-off (NMWCO) regenerated cellulose membrane (Pellicon 2, Millipore). The concentrate was stored at 4°C after addition of protease inhibitor (Sigma-Aldrich) at a final concentration of approximately 100 mg l^−1^ in an attempt to decrease viral degradation. The sample was then further concentrated with 100 kDa NMWCO Centricon-80 centrifugal ultrafiltration devices (Millipore) and stored at 4°C until fractionation.

### Viral Genome Size Distributions

Pulsed-field gel electrophoresis (PFGE) was used to monitor viral genome size distributions in the fractions collected from viral fractionation as an indicator of fractionation progress. Viruses in fractions were concentrated with 100 kDa NMWCO Nanosep centrifugal ultrafiltration devices (Pall) and processed for PFGE as previously described [24]. PFGE was carried out using a CHEF-DR II PFGE system (Bio-Rad) in Tris-Borate EDTA (TBE) buffer for 18 h with switch time ramping linearly from 1 to 12 s. DNA molecular weight markers (MidRange I and Lambda Ladder; New England Biolabs) and mass standards (High DNA Mass Ladder, Invitrogen) were run on all gels. Gels were stained overnight at 4°C with SYTO 60 (Invitrogen), then visualized and analyzed with the Odyssey Infrared Imaging System (Li-Cor Biosciences).

### Viral Fractionation

Continuous cesium chloride (CsCl) gradients were used as the first fractionation step to separate viruses from one another based on their differing buoyant densities [23]. The density of the viral concentrate was adjusted to 1.45 g ml^−1^ by the addition and dissolution of solid molecular grade CsCl (Fisher Scientific) and 10.5 ml of the resulting solution was deposited into a 12-ml polyallomer ultracentrifuge tube (Beckman Coulter). A 1-ml cushion of 1.52 g ml^−1^ CsCl that had been prepared with ultrapure water (NANOPure DIamond, Barnstead) and filtered through a 0.02 µm pore-size syringe filter (Acrodisc, Pall) was deposited at the bottom of the tube with a Pasteur pipet to avoid pelleting of viruses more dense than the initial solution density before the gradient formed. The gradient was then centrifuged at 25000 rpm for 72 hrs at 4°C with a swinging bucket rotor (SW 41 Ti, Beckman Coulter) in an Optima XL-80K ultracentrifuge (Beckman Coulter). Fractions of ∼500 µl were collected top down from the gradient using a fraction collector (Auto Densi-Flow, Labconco) on low speed. Density of the fractions was determined gravimetrically and viruses were enumerated in each fraction using epifluorescence microscopy [25] with the stain SYBR Gold (Invitrogen). Assuming an average DNA content of 55 ag per virus [26], the volume of fraction required to obtain 100 ng of viral DNA was prepared for viral genome fingerprinting.

A viscous whitish substance was observed in the completed CsCl gradient at densities >1.4 g ml^−1^. The distribution of genome sizes in fractions was the same in all fractions from this zone and similar to the unfractionated sample. Under the assumption that the viruses in this zone were aggregated or adsorbed to the unknown whitish substance, an attempt was made to desorb the viruses. The relevant fractions were pooled and Tween-80 (Fisher) was added at a final concentration of 1% followed by sonication of the sample for 3 minutes in a sonicator bath (Branson). The treated sample was then fractionated in a second continuous CsCl gradient.

A fraction from the continuous CsCl gradient was selected for further separation of viruses by strong anion-exchange chromatography [23]. A BioLogic HR Workstation (Bio-Rad) equipped with a 1-ml sample injector, gradient mixer, fraction collector, and UV and conductivity meters was used to run a step gradient through an UNO Q1 strong anion-exchange chromatography column (Bio-Rad). The starting buffer (20 mM Tris-HCl, pH 7.8) and elution buffer (20 mM Tris HCl, 1 M sodium chloride, pH 7.8) for chromatography were prepared with ultrapure water (NANOPure), autoclaved, and filtered through 0.22 µm pore-size filters. The remaining portion of the selected CsCl gradient fraction that had not been used for viral genome fingerprinting was exchanged into the chromatography starting buffer with a Centricon-20 centrifugal ultrafiltration device with a 100 kDa NMWCO filter (Millipore) and recovered at a final volume of ∼1.1 ml. The UNO Q1 chromatography column was equilibrated sequentially with 7 ml of starting buffer, 7 ml of elution buffer, and 7 ml of starting buffer at 1 ml min^−1^. The sample was then loaded onto the column and a step gradient was run with 1% steps of increasing elution buffer between 26 and 42% elution buffer at 0.5 ml min^−1^, with collection of 8 ml fractions per step. For each fraction, 300 µl was used for viral genome fingerprinting and the remaining volume was stored at 4°C. A fraction from this gradient was then selected for analysis with transmission electron microscopy (TEM), shotgun clone library construction, and sequencing.

### Transmission Electron Microscopy

The morphological diversity of viruses in the selected fraction was investigated with TEM. An air-driven ultracentrifuge (Airfuge CLS, Beckman) was used to deposit viruses from 200 µl of the fraction on to copper grids (200 mesh) with carbon-stabilized formvar that had been rendered hydrophilic by UV irradiation (240 mJ). The grids were secured to the distal interior surface of the Airfuge rotor chambers (EM-90, Beckman) and the sample was centrifuged for 20 minutes at 118 000× *g*. Viruses on the grid were then stained with 10 µl of 0.02 µm-filtered 2% uranyl acetate for 45 s. The stain was then wicked away with absorbent filter paper (Whatman) and the grids were rinsed with 10 µl of ultrapure water (NANOPure DIamond, Barnstead) which was also wicked away with absorbent filter paper. The stained grids were then air dried and stored desiccated at room temperature (18–24°C) until analysis. Grids were examined at 100 000–125 000× magnification using a transmission electron microscope (LEO 912) with 100 kV accelerating voltage. Micrographs were taken of the first 50 observed viruses with a Proscan Slow-Scan Frame-Transfer cooled CCD camera with 1K ×1K resolution run with analySIS software (Soft Imaging Systems). Image-Pro Plus software (Media Cybernetics) was used to measure the capsid diameters and tail lengths of the first 50 observed viruses.

### Library Construction and Sequencing

Viruses in the remaining portion of the fraction were concentrated with a 100 kDa NMWCO Nanosep centrifugal ultrafiltration device (Pall) and the DNA was extracted with a MasterPure Complete DNA and RNA Purification Kit (Epicentre). The extracted DNA was then split into four samples and separate clone libraries were constructed from three of the extracted samples. The DNA in those samples was amplified with three separate multiple displacement amplification (MDA) reactions (REPLI-g, Qiagen) in an effort to reduce amplification bias as a result of MDA [27]. After extracting the amplified DNA, one of the samples was then physically sheared to 3–5 kb using a HydroShear (Genomic Solutions) while the other two samples were sheared to 1–2 kb. The sheared samples were then purified with a MinElute PCR Purification Kit (Qiagen), the ends were made blunt with a DNA Terminator End Repair Kit (Lucigen), and gel electrophoresis was used to isolate the appropriate sizes of DNA from each sample. DNA was extracted from the first sample in the gel with a MinElute Gel Extraction Kit (Qiagen), but this resulted in low recovery of the DNA (∼5%), so the other two samples were extracted from the gel with a Centrilutor micro-eluter (Millipore), resulting in 35 to 52% recovery. A clone library was then constructed from each of the samples using the CloneSmart Blunt Cloning Kit (Lucigen). Plasmid sequencing of the clones from the three libraries was conducted with dye-terminator Sanger sequencing at the University of Hawai‘i Advanced Studies in Genomics, Proteomics, and Bioinformatics sequencing facility. Paired-end reads were obtained from 391 of the 1651 sequenced inserts for a total of 1942 sequences.

### Analysis of Sequences

Sequences from the 3 libraries were pooled and analyzed as one library. Sequence trimming and assembly were performed with Sequencher 4.10.1 (Gene Codes Corp.). Vector sequence was removed using the automatic recognition function in the software. Assembly of all sequences to the vector sequence as a template revealed additional vector-only sequences, which were removed. Forward and reverse reads of the same clone were assembled using the “Assemble by Name” function. Some of these assemblies produced odd results, with forward and reverse reads in same direction. In some cases, the second strand assembled to the first immediately after a string of Ns in the middle of the first strand. These odd assemblies (11 contigs of 22 sequences) were removed. The remaining sequences were trimmed such that the first and last 99 base pairs (bp) contained <1 ambiguity and the first and last 20 bp contained <2 bp with a confidence value <40%. These conditions were applied repeatedly until all sequences met the criteria. The sequences were then trimmed further using the criteria that the first and last 20 bp had <1 bp with a confidence <20%. In some cases, sequences with poor quality regions (strings of Ns) in the middle of the sequence were not identified by these criteria and these were trimmed by hand to remove all sequence at and following the ambiguous bases. After trimming, sequences of <100 bp were removed leaving 1796 unassembled sequences. These sequences were deposited in GenBank (Accession Numbers JS807804–JS809599).

Sequences in the library were compared to the GenBank non-redundant protein database using BLASTx [28], [29], omitting sequences from uncultivated organisms. The sequences were classified based on the identity of the sequence with which it shared the greatest similarity, except when the most similar sequence was non-viral, but the sequence also displayed significant similarity (E-value ≤0.001) to a virus. In the latter case, the sequences were classified according to the most similar virus-derived sequence. Sequences classified as viral were further classified based on their family and protein type.

### Phylogenetic Analysis

In an effort to assess phylogenetic diversity of viruses in our library, sequences that had any significant similarity (not just the highest similarity) to a viral DNA polymerase were used to construct a phylogram. These sequences were translated and aligned with other translated DNA polymerase gene sequences from viral genomes present in GenBank using custom scripts. A maximum-likelihood tree was then constructed based on this amino acid alignment as previously described [30] with RAxML [31] using the WAG substitution matrix with a subset estimation of invariable sites and gamma distribution in four discrete categories (WAG+Γ_4_+ I).

### Sequence Assembly and Contig Analysis

Sequencher was used to assemble forward and reverse reads using the “Assemble by Name” function. Those that assembled were merged into consensus sequences. The resulting 1723 sequences were then assembled using the criteria of a minimum overlap of 20 bp and a minimum of 98% identity according to Breitbart et al. [13]. Open reading frames (ORFs) were predicted in only the larger assembled contigs (>4 kb) using GeneMark.hmm 2.0 [32] and annotated by comparing the ORF sequences to the GenBank non-redundant protein database using BLASTx [28], [29] with the same criteria used as when analyzing the trimmed sequence library.

## Results

### Viral Fractionation

**Figure 1:**
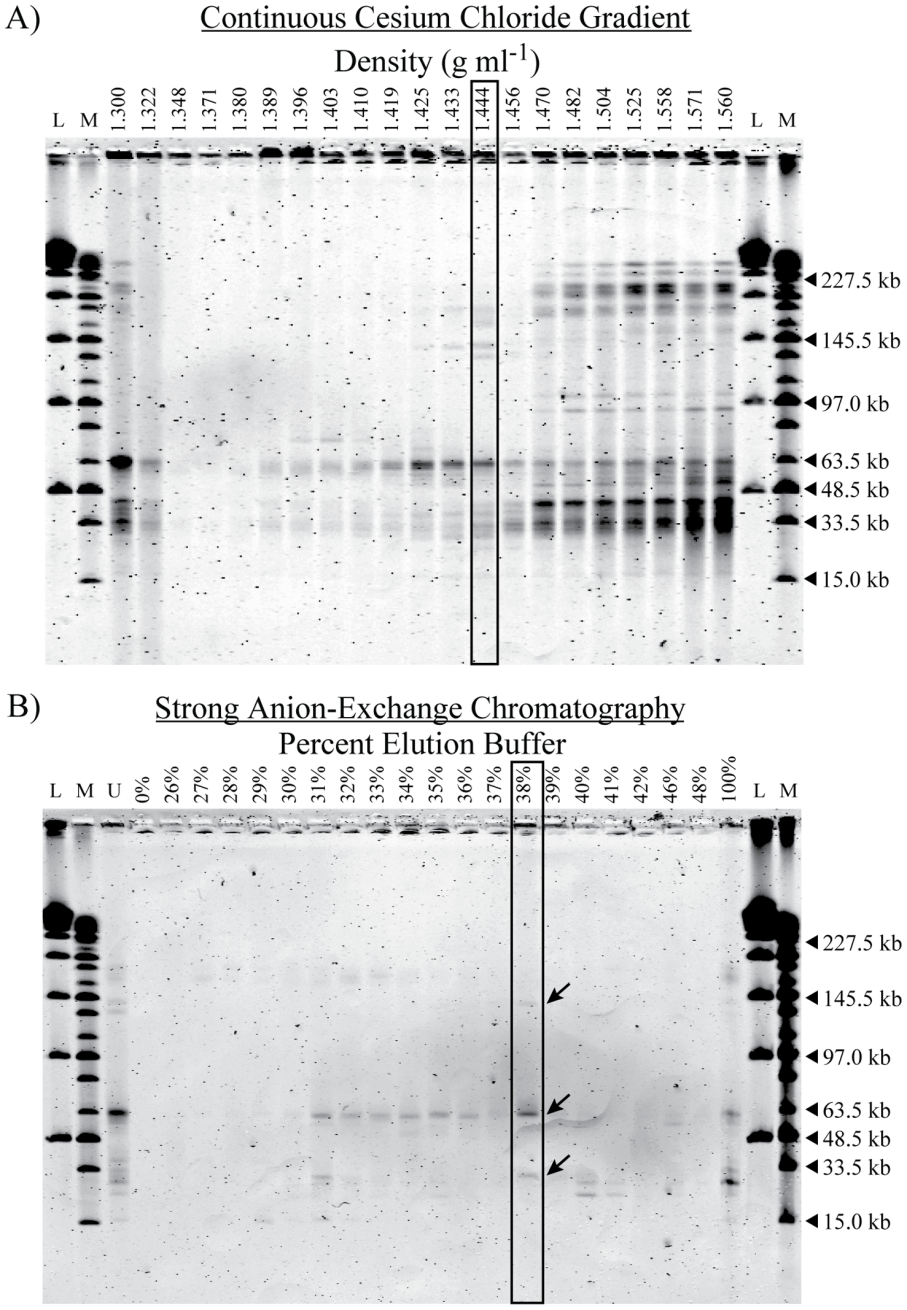
Viral genome fingerprints of the fractions used in each fractionation step. (A) Pulsed-field gel of the virus assemblages in each fraction collected from a continuous cesium chloride gradient of a viral concentrate from Kāne‘ohe Bay. The box around the fraction with a density of 1.44 g ml^−1^ indicates that fraction was separated further using strong anion-exchange chromatography. (B) Pulsed-field gel of the virus assemblages in each fraction collected from the further separation of the indicated cesium chloride gradient fraction using strong anion-exchange chromatography. The box around the fraction that eluted with 38% elution buffer indicates the fraction selected for microscopy and sequencing. Arrows point to the three genome bands in the fraction. Marker lanes contain a Lambda Ladder (L) and a MidRange PFG Ladder (M). The unfractionated sample was also run for comparison (U).

In the initial continuous cesium chloride (CsCl) gradient of the viral concentrate, a large portion of the viruses banded with little resolution over a broad range and at high densities (1.47–1.56 g ml^−1^; data not shown), an atypical result for this method [23]. The presence of a viscous whitish matter in this region of the gradient suggested that the viruses could be adsorbed to an unknown substance. After treatment of all pooled fractions with Tween-80 and sonication, followed by separation in a second gradient, much of the material banded in the same position and remained unresolved (Figure 1A). There was also an aggregation of viruses that banded at the top of the gradient (1.300–1.322 g ml^−1^). The remaining viruses were found in nine fractions between 1.389 and 1.456 g ml^−1^ and most of these fractions showed distinct patterns of genome sizes.

Viruses in the fraction having a density of 1.444 g ml^−1^ were subjected to a second round of fractionation by anion-exchange chromatography. Most of the viruses eluted in 11 of the 21 fractions between 31% and 46% elution buffer with the gradient ending at 48% (Figure 1B). A final rinse out with 100% elution buffer resulted in the release of additional viruses, most likely those adsorbed to the unknown substance. The fraction that eluted with 38% elution buffer was selected for sequencing and included three visible viral genome bands. The dominant band was 62 kb and included 65% of the DNA in the fraction. The two minor bands were 31 kb and 139 kb and included 18% and 17% of the DNA in the fraction, respectively.

### Transmission Electron Microscopy

**Figure 2:**
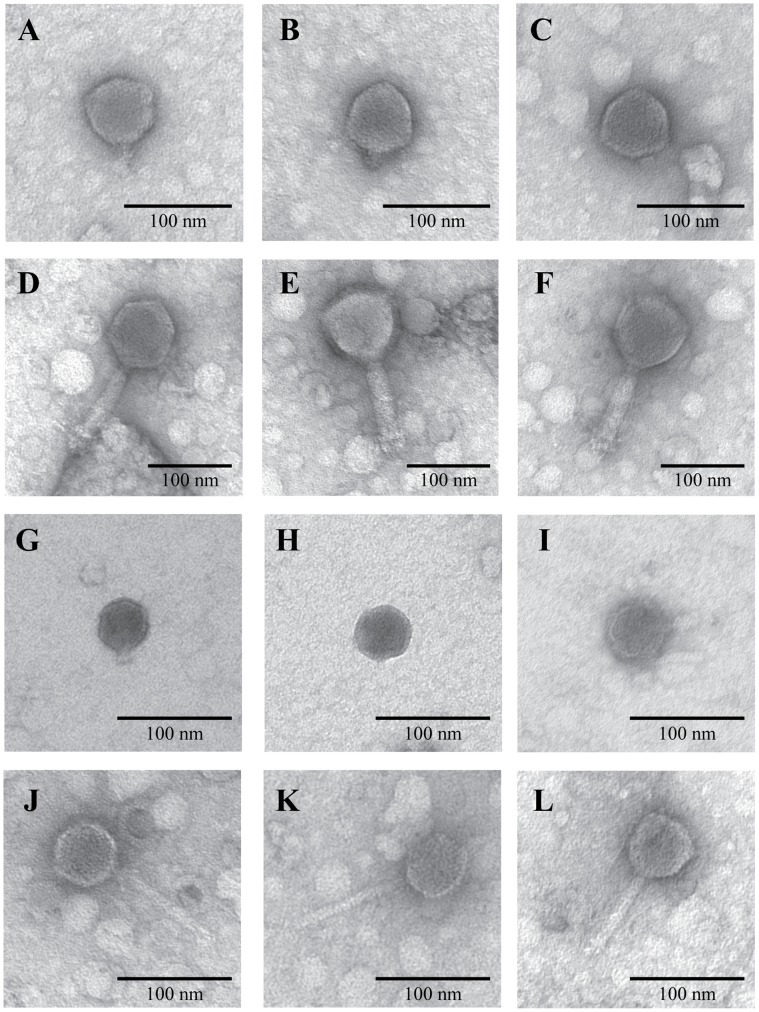
Transmission electron micrographs of viruses in the fraction selected for sequencing. Representative viruses from the four morphological groups in the fraction are shown in A–C, D–F, G–I, and J–L. These groups comprised 44, 30, 19, and 7% of the population, respectively.

Analysis of the viruses in the selected fraction with transmission electron microscopy (TEM) revealed that the fraction had four readily distinguishable morphotypes. The dominant morphotype, which comprised 44% of the population, had podovirus morphology with capsid diameters between 60 and 67 nm, and short (14–18 nm) tails or no visible tail (Figure 2A–C). The second group of viruses, which comprised 30% of the population, had myovirus morphology with capsid diameters between 76 and 103 nm, and long (109–118 nm) contractile tails (Figure 2D–F). The third group of viruses, which comprised 19% of the population, had podovirus morphology with capsid diameters between 44 and 50 nm, and short (15–17 nm) tails or no visible tail (Figure 2G–I). The fourth group of viruses, which comprised 7% of the population, had siphovirus morphology with capsid diameters between 52 and 60 nm, and long (100–102 nm) non-contractile tails (Figure 2J–L).

### Sequence Composition

**Figure 3:**
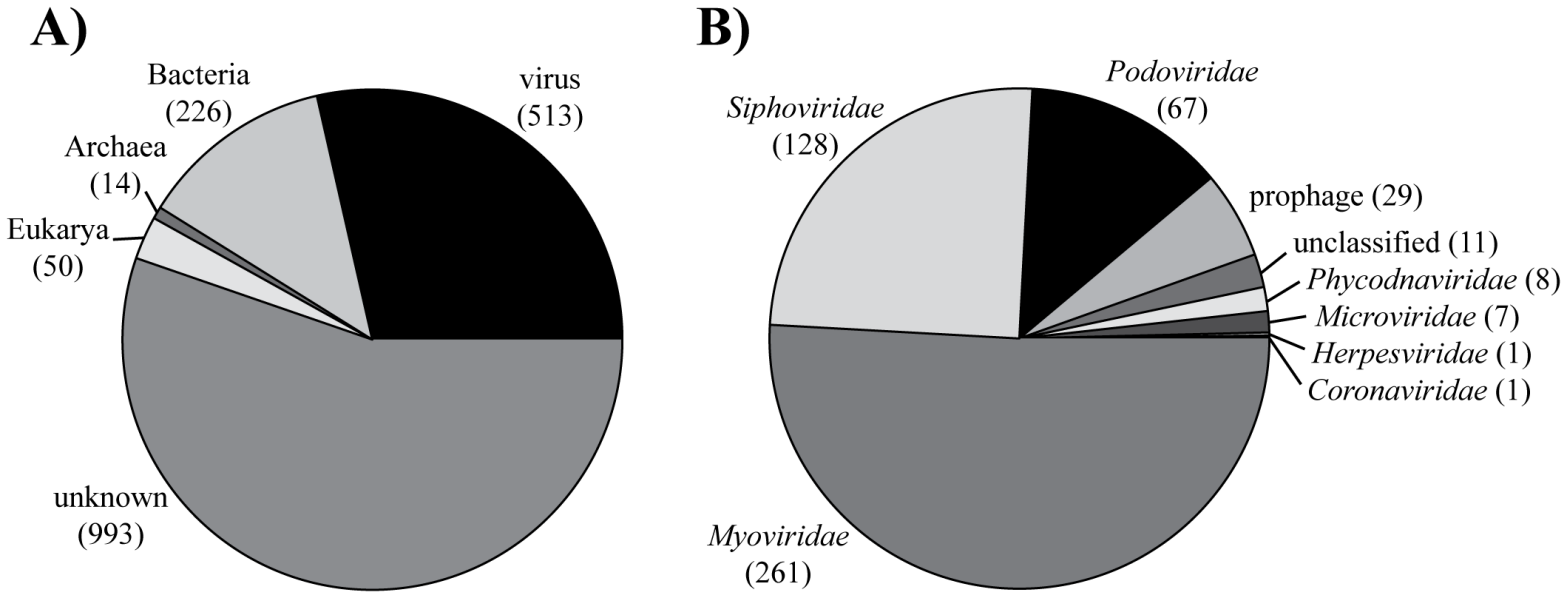
Taxonomic classification of the sequence library. Classification of all sequences (A) and families represented in the virus sequences (B) based on significant hits (E-value ≤0.001) to the GenBank database using BLASTx. Numbers of sequences are in parentheses.

After trimming, the average read length in the library was 609 (±130) bases and the average G+C content was 36 (±5)%. A search in the GenBank database using BLASTx revealed that the majority (55%) of sequences in the library had no significant similarity to other deposited sequences, 28% were similar to sequences from viruses, 13% to sequences from bacteria, and 4% to sequences from eukaryotes and archaea (Figure 3A). Of the virus-like sequences, 51% were similar to sequences derived from myoviruses, 25% to sequences from siphoviruses, and 13% to sequences from podoviruses (Figure 3B). The viruses from which nearly all of these most similar sequences derived were bacteriophages including three *Synechococcus* phages, three *Pseudomonas* phages, and two *Prochlorococcus* phages (Table 1). Matches to virus-derived genes included oxygenases, helicases, structural proteins, and DNA polymerases, but nearly half (47%) were to genes with unknown function (Table 2).

**Table 1:** Viruses in the GenBank database with the highest number of significant similarities from the sequence library.

Virus	Percent of total virus hits
*Synechococcus* phage S-SM1 (myovirus)	14.8%
*Pseudomonas* phage YuA (siphovirus)	7.7%
Bacteriophage phiJL001 (siphovirus)	6.7%
*Pseudomonas* phage LUZ24 (podovirus)	6.0%
*Synechococcus* phage S-PM2 (myovirus)	4.8%
*Synechococcus* phage syn9 (myovirus)	4.2%
*Pseudomonas* phage M6 (siphovirus)	3.8%
*Prochlorococcus* phage P-SSM2 (myovirus)	3.1%
*Prochlorococcus* phage P-RSM4 (myovirus)	2.9%
*Vibrio* phage VP2 (podovirus)	2.7%

**Table 2:** Categories of viral proteins in the sequence library.

Protein category	Number of sequences
unknown	245
oxygenase	63
helicase/primase	49
structural	37
DNA polymerase	31
exonuclease	25
ferrochelatase	21
DNA synthesis	13
peptidase	9
DNA packaging	5
DNA methylase	3
integrase	3
endolysin	2
endonuclease	2
DNA binding	1
heat shock protein	1
protease	1
transcriptional activator	1
transferase	1

### Phylogenetic Analysis

**Figure 4:**
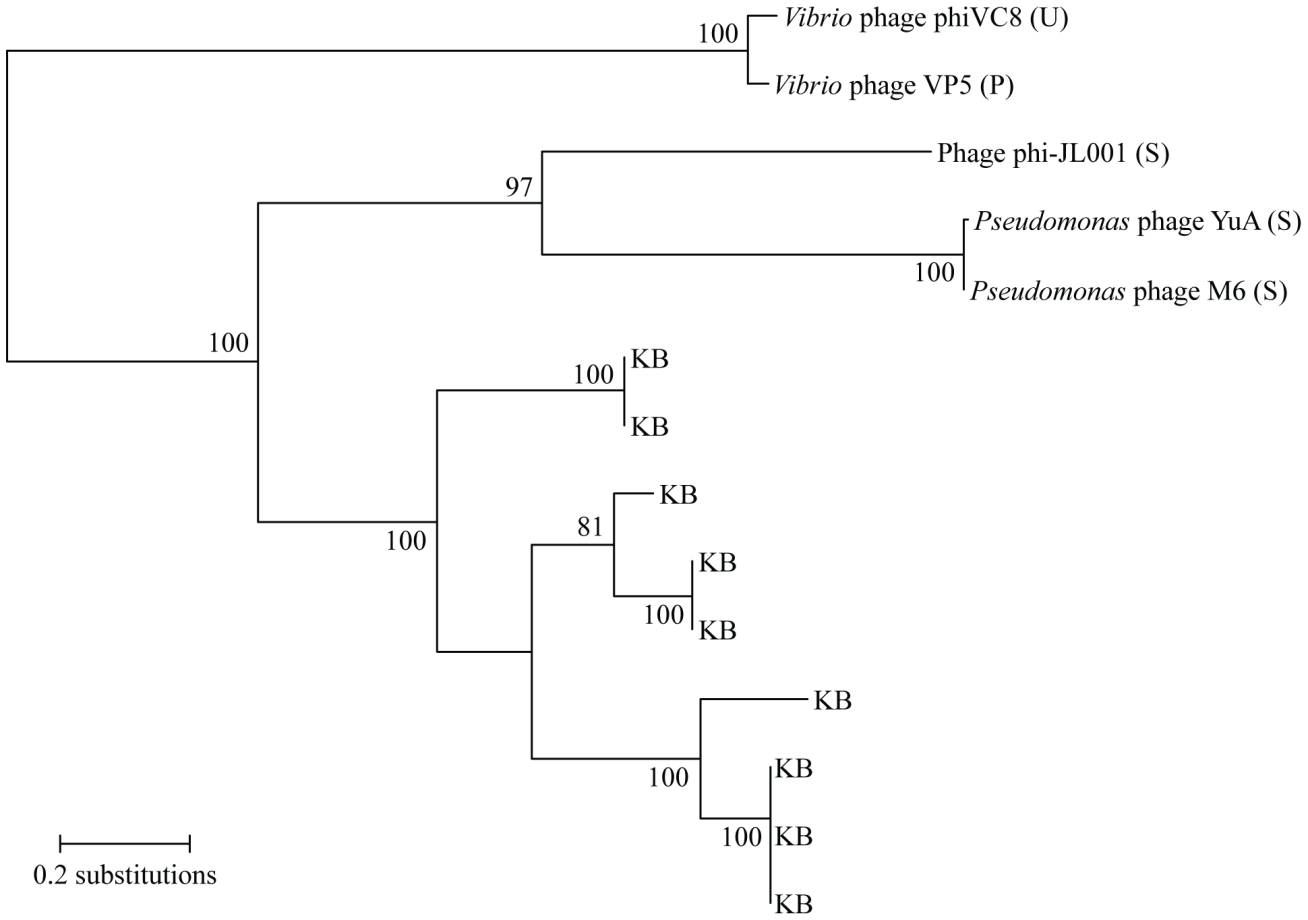
Phylogenetic evaluation of DNA polymerase sequences in the sequence library. The unrooted phylogenetic tree was based on a 96 amino acid residue region of viral DNA polymerase sequences obtained from GenBank and putitive DNA polymerase sequences from this study. The letter designations P, S, and U correspond to *Podoviridae*, *Siphoviridae*, and unclassified viruses, respectively. All sequences from the Kāne‘ohe Bay library are designated with KB. Bootstrap values based on 100 resamplings are shown at the nodes if they were >50.

Fifty sequences in the library had significant similarity to viral DNA polymerases, with 34 of the sequences having the greatest similarity to the DNA polymerase of bacteriophage phi-JL001 [33]. An alignment of 9 of these sequences across 96 amino acid residues of the conserved DnaQ-like region of the polymerase, as determined using the Conserved Domain Database [34], was used to construct a phylogenetic tree (Figure 4). Although there was deep-branching support for clustering of the library sequences with the siphoviruses phi-JL001, YuA, and M6 (bootstrap value 100), the sequences from our Kāne‘ohe Bay library formed their own well-supported clade (bootstrap value 100) with five groups.

### Sequence Assembly and Contig Annotation

**Figure 5:**
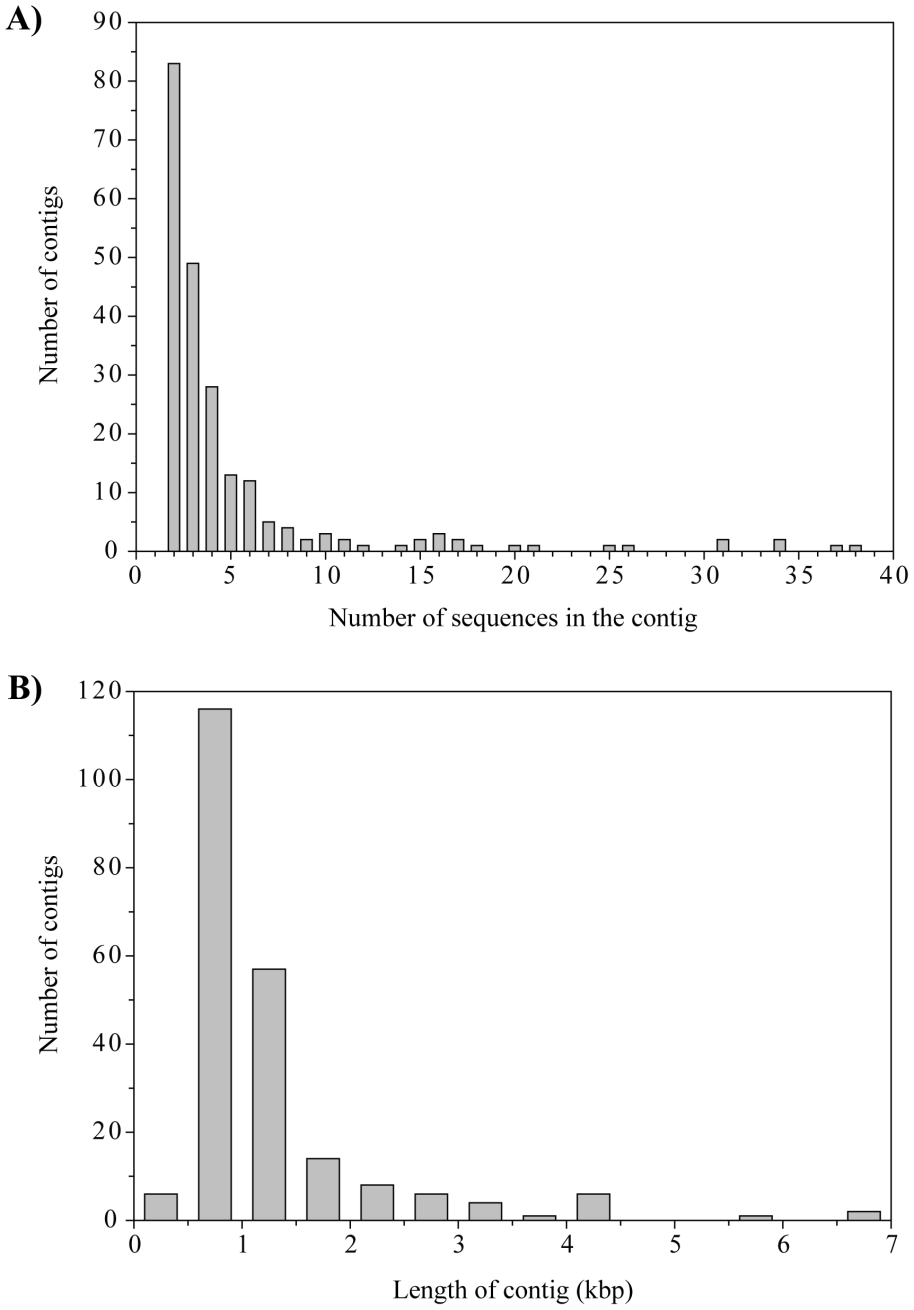
Contig spectrum and length distribution of contigs assembled from the sequence library. (A) Histogram of the number of sequences in each contig assembled with Sequencher using conditions of 98% minimum match and >20 bp overlap. (B) Histogram of the lengths of those contigs.

**Figure 6:**
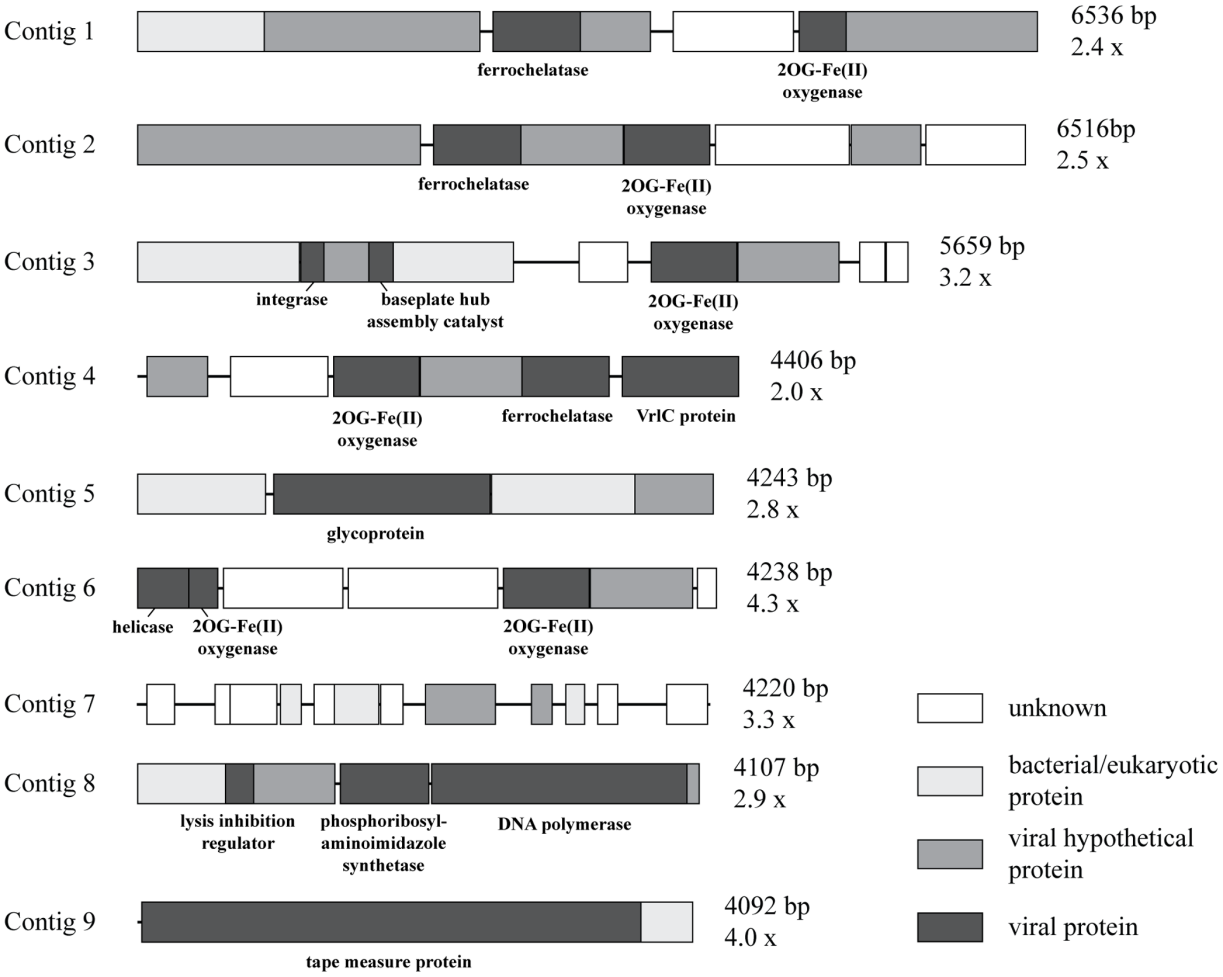
Annotation of ORFs in large contigs (>4 kb) assembled from the sequence library. Length and coverage of each contig are listed at right.

Assembly of the sequences resulted in 221 contigs comprised of 2 to 38 sequences each (Figure 5A) and ranging in size from 370 to 6536 bp in length (Figure 5B), with 65% of the sequences in the library comprising these contigs. Identification of ORFs in the largest contigs (>4 kb) revealed 47 complete ORFs with an average length of 640 bp (Figure 6). The majority of these contigs had larger ORFs, but the seventh contig was comprised entirely of short ORFs (111–513 bp) with no significant hits and the ninth contig contained a much larger ORF (3672 bp) with similarity to a viral tape measure protein. Annotation of the ORFs showed that they were primarily composed of viral sequences including repeated, highly significant hits (E-value <10^−19^) to ferrochelatase and 2OG-Fe(II) oxygenase genes from the *Synechococcus* phage S-SM1 [35].

## Discussion

PFGE and morphological analyses supported the hypothesis that physical fractionation of a viral assemblage from Kāne‘ohe Bay could be used to enrich a limited number of viruses in a fraction. PFGE analysis indicated the presence of three distinct genome sizes, while TEM showed four distinct morphological groups. Both PFGE and TEM can underestimate actual diversity, since genetically distinct viruses can have indistinguishable genome sizes [24] or morphologies [36]. Given these caveats, we found that there was a minimum of four distinct groups of viruses in the sequenced fraction.

The sequence library did not contain matches to more than a few genes of any one virus, suggesting that the viral genomes represented in the library have not previously been sequenced. Most virus hits were to bacteriophages, consistent with the observed morphologies of the viruses in the sample, which mostly resembled tailed bacteriophages in the order *Caudovirales*.

The distant relationships of our library sequences to known viral DNA polymerase sequences suggest that the viruses in the sequenced fraction are not closely related to any previously sequenced virus, and thus information about their potential hosts cannot be inferred from the phylogenetic tree. However, the library sequences formed a well-supported clade, suggesting that the viruses in the fraction used to construct the library were relatively closely related with respect to the phylogeny of their putative DNA polymerase sequences. The phylogenetic results also show that there were viruses belonging to at least five operational taxonomic units in the sequenced fraction.

While we did not directly compare the fractionated viral assemblage to the whole, unfractionated viral community, assembly of the sequence library from the fractionated sample showed that there were many more contigs generated than from comparable metagenomic analyses of whole viral assemblages [11]–[13], [37], [38]. In the latter studies, only 0.3–3.5% of library sequences could be assembled into contigs with a maximum of 4 sequences per contig, whereas 65% of the sequences in our library were assembled into contigs with a maximum of 38 sequences in a contig. This supports the hypothesis that, by physically fractionating viral assemblages, there will be significantly greater reassembly of sequences from libraries constructed with the resulting fractions [21], [22].

The longer contigs assembled from this fractionated viral assemblage allowed for an assessment of genes within the context of genomic fragments from uncultivated viruses. ORFs with high similarity to 2OG-Fe(II) oxygenase were found in five out of the nine analyzed contigs. This gene has so far been found exclusively in T4-like cyanophages [35], suggesting that these five contigs came from the genome of the myovirus identified in the fraction. The fact that these genes occurred in multiple contigs, but in different locations relative to other genes, indicates that there could be several types of morphologically similar myoviruses with different genome arrangements in our sequenced fraction. Alternatively, these similar contigs could be chimeric assemblies resulting from low sequence coverage (2.0–4.3x), chimeras generated from MDA [39], or both.

Although we used a large volume concentrate for this study, this is not required to take advantage of the fractionation approach. Our motivation for using a large volume was to ensure that we had sufficient material to document separation at each stage using PFGE. We also anticipated that with sufficient starting volume, we might be able to avoid amplification of the material before cloning. Direct cloning would have been possible for some of the fractions, but the one we chose for analysis did not have sufficient material. The MDA amplification step we employed has been used in other marine viral metagenomes (e.g., [14]), but can result in biases [27], [40] and the formation of chimeras [39]. Such problems may explain some of the odd forward and reverse assemblies noted in the materials and methods and the repetition of genes within a contig. The increased assembly we achieved through fractionation and the long reads from Sanger sequencing make these problems more apparent. The use of improved amplification methods [41] or elimination of the amplification step [38], coupled with increases in sequencing power [20], should further improve our ability to accurately reassemble the genomes of uncultivated viruses isolated by physical fractionation. This is a worthwhile goal, because with accurate genome reassembly, one can move beyond metagenomic gene inventories and conduct comparative genomics of uncultivated viruses.

There are other methods for more efficiently assembling viral genomes from complex assemblages, such as the use of large-insert clone libraries [42], [43] or single-virus amplifications [44]. These methods are also fractionations, but rely on fractionation to the level of single genomes or virions. Bulk fractionation offers significant, complementary advantages. By fractionating populations of intact viruses en masse, it is possible to enrich for even rare populations of interest by screening with specific primers at each stage of the separation. Further, by narrowing the target populations while maintaining sufficient numbers of intact virions, it also becomes possible to more clearly link viral genomes with proteomes and with the physical properties of the virions (buoyant density, surface charge, morphology). Thus, we propose that an effective way to advance our understanding of uncultivated viral populations will be to combine the advantages of bulk fractionation with other methods that allow the assembly of discrete genomes. Initial bulk physical fractionation of a community will allow targeted separation and phenotypic characterization of populations, and subsequent single-virus genomics (whether by amplification, large-insert cloning, or direct sequencing) performed on a portion of the fractionated populations will allow accurate genome assemblies of the phenotypically characterized populations.
